# Supplementary material for: NELL2-Robo3 complex structure reveals mechanisms of receptor activation for axon guidance
Source: Nat Commun. 2020 Mar 20;11:1489. doi: 10.1038/s41467-020-15211-1 (PMC7083938; doi:10.1038/s41467-020-15211-1)
Supplement: Supplementary file 1 — Supplementary Information [file 41467_2020_15211_MOESM1_ESM.pdf]

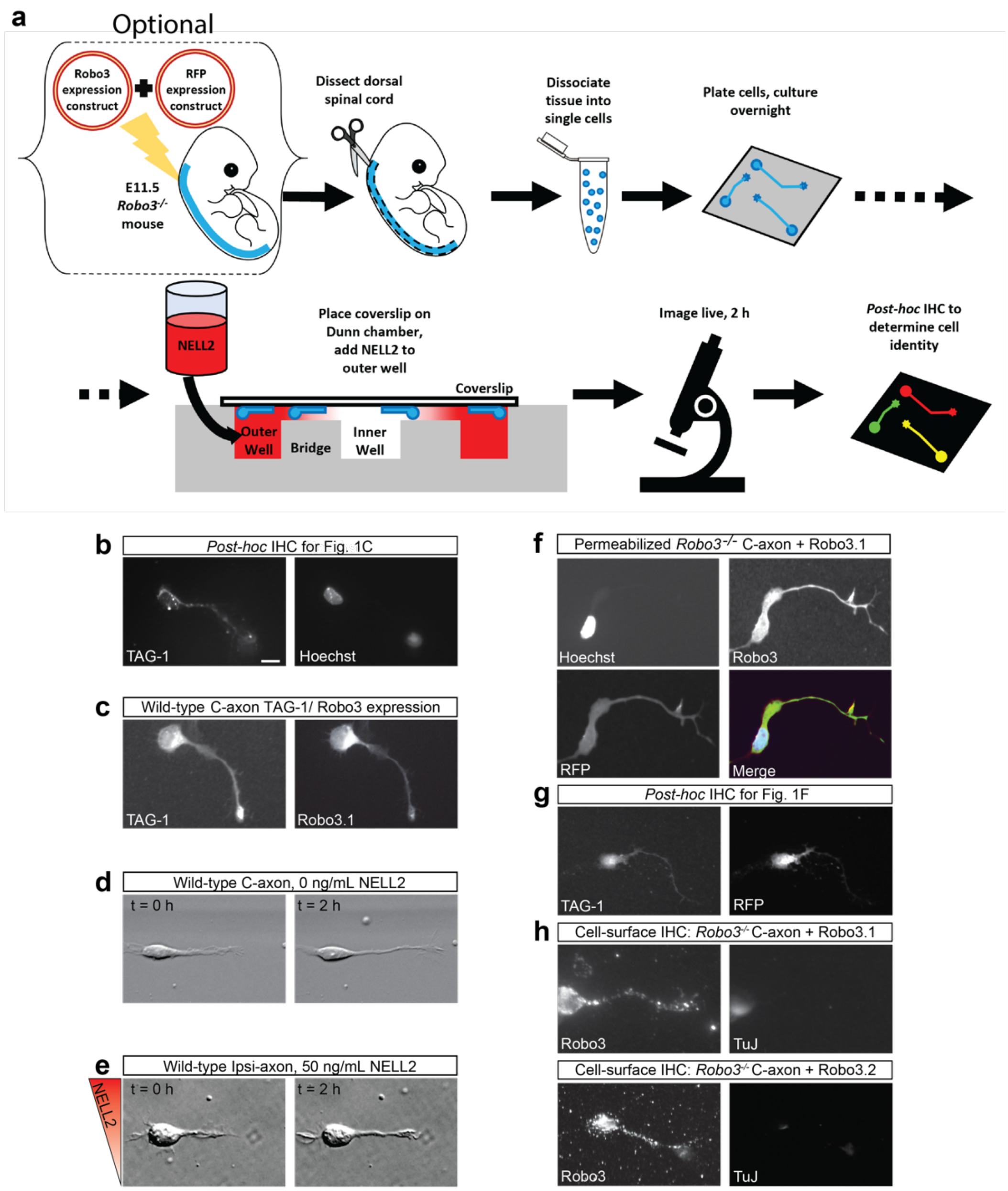

Supplementary Fig. 1. NELL2 Dunn chamber axon turning assays. (a) Schematic of Dunn chamber turning assay experimental flow. **(b)** *Post-hoc* immunolabeling of a TAG-1-positive commissural neuron after exposure to NELL2 in the Dunn chamber. **(c)** Double immunolabeling of a commissural neuron shows TAG-1 and Robo3.1 co-expression. These two markers coincide in 100% of cultured commissural neurons. **(d)** DIC images of a commissural neuron cultured in the absence of NELL2 (0 h and 2 h) shows no turning. **(e)** DIC images of dorsal spinal cord ipsilaterally projecting neuron shows no NELL2-induced axon turning. **(f)** Immunolabeling of a Robo3.1-electroporated *Robo3*$^{-/-}$ commissural neuron shows co-expression of exogenous Robo3.1 and RFP. **(g)** *Post-hoc* immunolabeling of a TAG-1-positive *Robo3*$^{-/-}$ commissural neuron electroporated with RFP and Robo3.1 after exposure to NELL2 in the Dunn chamber. **(h)** Non-permeabilized immunolabeling with a Robo3 ECD antibody demonstrates Robo3.1 (top) and Robo3.2 (bottom) expression on the surface of *Robo3*$^{-/-}$ axons after electroporation. Scale bar, 10 µm (b-h).

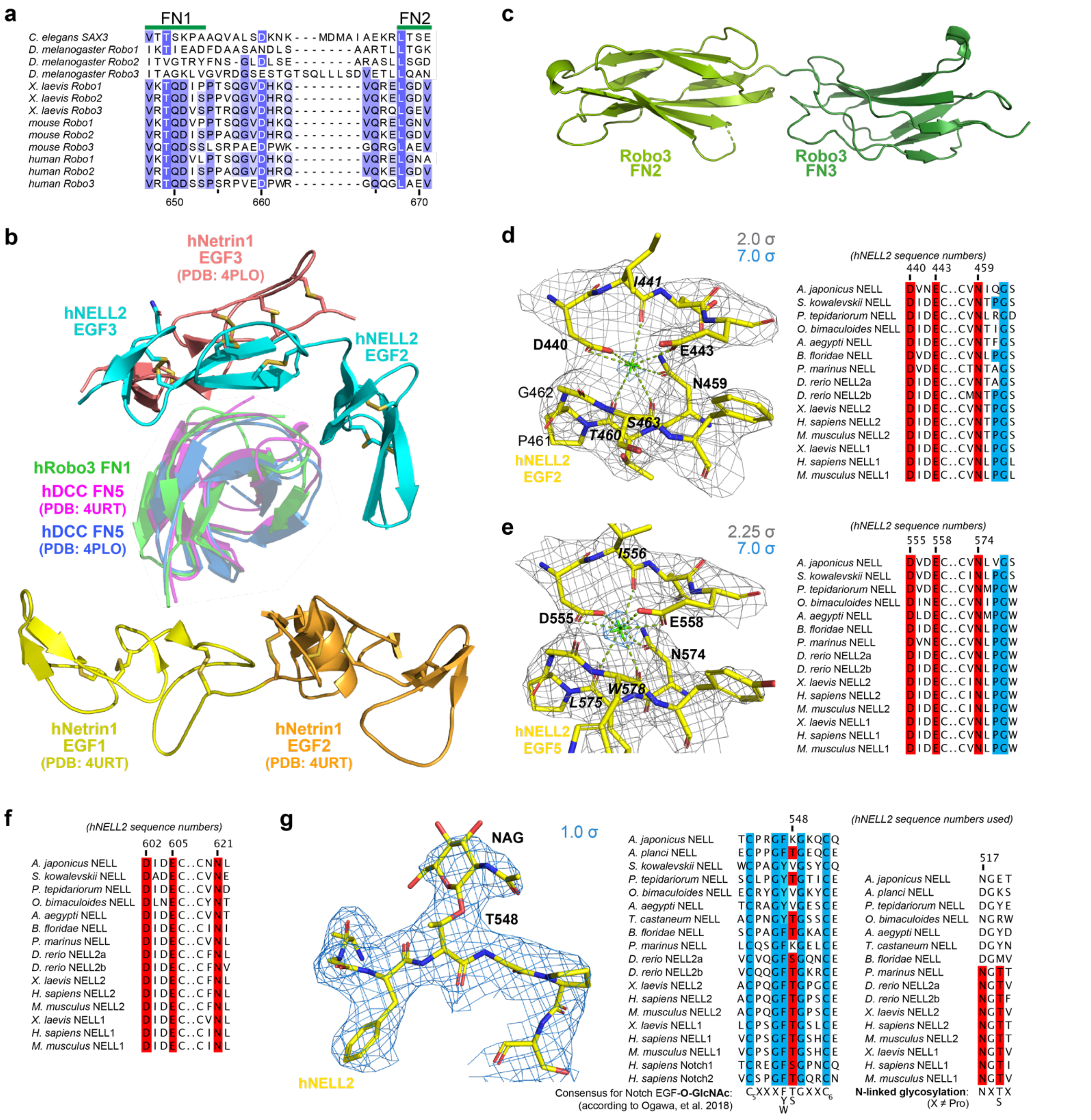

Supplementary Fig. 2. Detailed structural features of Robo3, NELL2 and their complex. (a) Sequence alignment of the protease-sensitive linker region between FN1 and FN2 domains of Robos. The ~15-amino acid linker is present but not conserved in invertebrates. The sequence numbering below the alignment is for hRobo3. The green lines represents amino acids observed clearly in electron density maps in the NELL2-EGF1-6+Robo3-FN1 and Robo3-FN2-3 structures. **(b)** The crystal structure of hRobo3 FN2 and FN3 domains. **(c)** Robo3-NELL2 FN-EGF complex adopts an entirely different geometry compared to Netrin-DCC FN-EGF complexes. The FN domains of hRobo3 (FN domain 1) and hDCC (FN domain 5) are aligned at the center of the image. **(d,e)** Electron density for the two calcium ions bound to hNELL2 EGF2 (d) and EGF5 (e) domains at sites conserved among vertebrate and invertebrate NELL sequences. Two contour levels were used to draw electron density, where the lower cutoff shows protein density and the higher cutoff only shows the electron-

rich calcium ions. A likely third calcium ion bound to EGF6 is not well resolved due to flexibility of the EGF6 domain. Bold residue labels indicate amino acids with side chains coordinating calcium. Bold and italic labels indicate amino acids that coordinate calcium with main chain carbonyl oxygens. Additionally, two amino acids, a Proline and a Glycine, help reorient the main chain for the formation of the calcium coordination sites and are generally conserved. **(f)** Both O- and N-linked glycosylation is observed in the NELL2-Robo3 structure. (Left) The electron density of the O-linked glycan residue on the EGF4 domain (T548 in hNELL2) is drawn at 1 σ. (Middle) Consensus of the Notch EGF O-linked glycan aligned to NELL1 and NELL2 sequences. The site is present in vertebrate and some invertebrate NELLs. The expression system we used for NELLs is lepidopteran, which shows that arthropods can specifically add the EGF-specific O-linked N-acetylglucosamine. (Right) N-linked glycan residue at N517 (hNELL2) is conserved in vertebrates, but is absent in invertebrates.

a

	K_D (μM)
WT hNELL2 EGF	0.77
R448A/Y450A	9.0
R452A	1.1
D476A	~50
D477A/Y478A	≥100
L498A/F500A	≥100
V509A	1.5
WT hRobo3 FN	0.89
T604A	7.9
E639A	1.1
R630A	≥100
W635A	2.9
N600A/T601A	2.9
F594A/W602A	≥100
I624A/L626A	6.5

Apparent K_D for cell staining experiments (Fig. 2f,g).

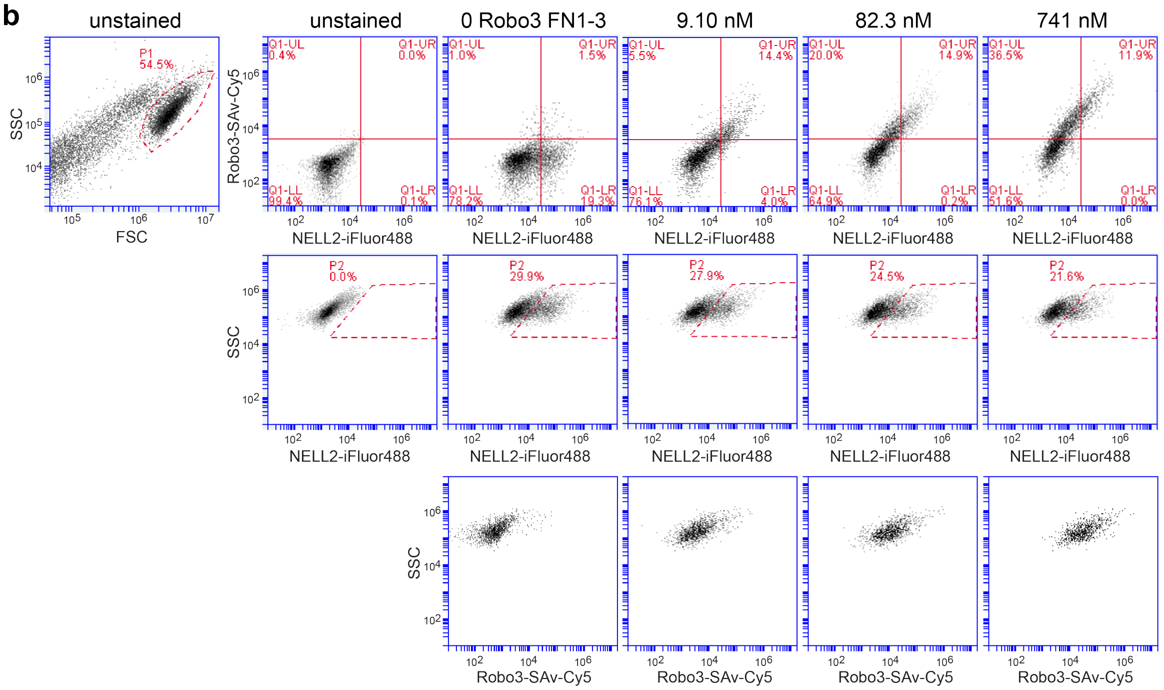

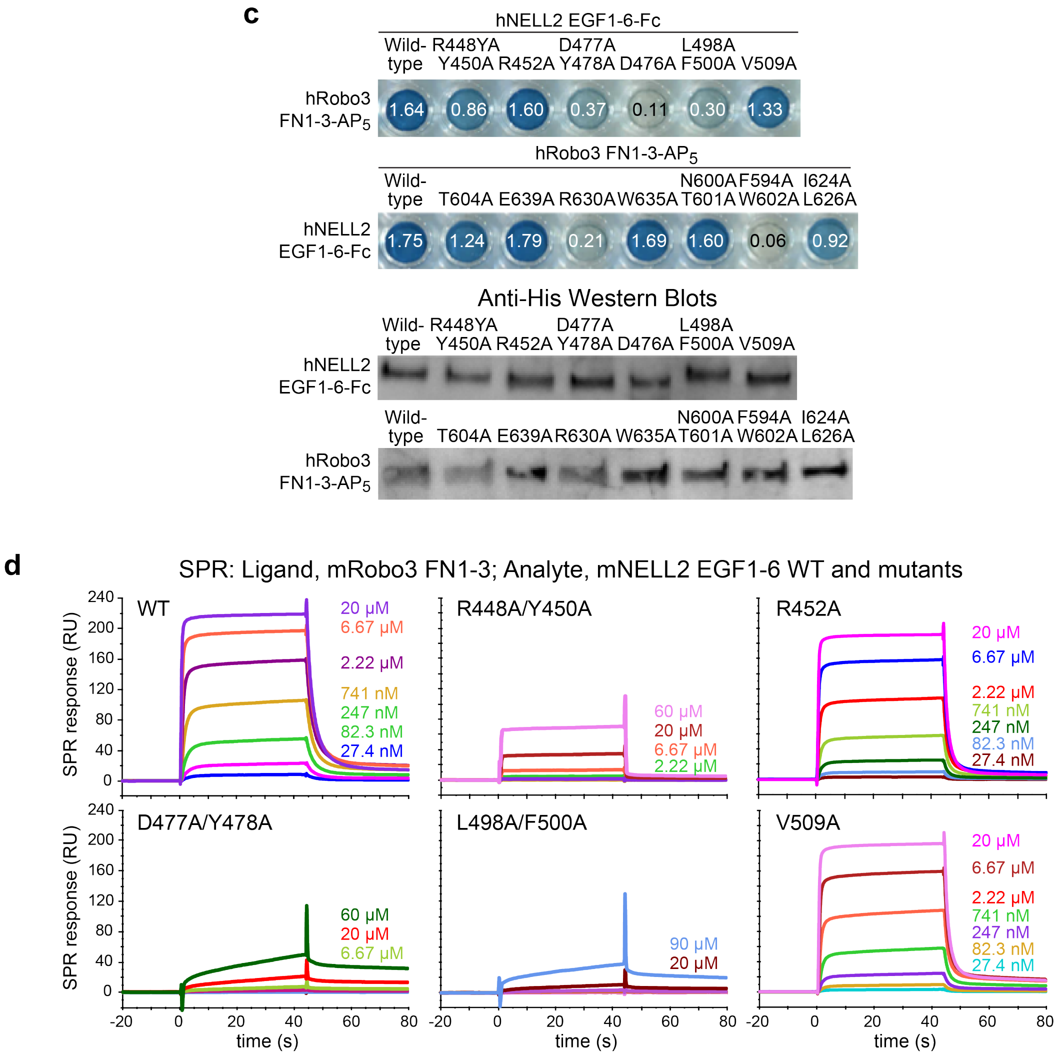

Supplementary Fig. 3. Mutational analysis of the NELL2-Robo3 interface. (a) Apparent dissociation constants measured by the S2 cell staining experiments in Figs. 3d and 3e, calculated with a single-site binding

model in Prism 6 (GraphPad). WT: Wild-type. **(b)** Representative flow cytometry results for C-terminally FLAG-tagged NELL2 EGF1-6 expressed on S2 cell surface stained with Robo3 FN1-3-biotin and SAv-Cy5. The gating on FSC-SSC log-log plot (labeled P1) to remove cellular debris is shown for unstained control. A second gating (labeled P2) is applied to select for NELL2-expressing cells. Finally, MFI levels on the Robo3-SAv-Cy5 axes are used to plot Robo3 binding to NELL2. **(c)** (Top) ECIA results of engineered single and double point mutants of hNELL2 EGF1-6 and hRobo3 FN1-3 at the interface. hNELL2 was fused with an Fc tag (bait) and hRobo3 was fused with a pentamerized AP (prey). Blue color indicates the presence of prey, i.e. binding, which was quantified by absorbance at 650 nm (shown numerically in each well). (Bottom) Protein quantities were normalized within each set of WT and mutant constructs using fluorescent signal from western blots using an anti-his-tag antibody coupled with the fluorophore iFluor 488. **(d)** SPR sensorgrams for the interaction of mRobo3 FN1-3 with mNELL2 EGF1-6 WT and mutants. Each color in the sensorgrams represents the concentration of the analyte in mobile phase. Zero time-point indicates time of analyte injection. mRobo3 FN1-3 was captured on a Biacore SA (streptavidin) chip, and titration series of hNELL2 EGF1-6 WT, R448A/Y450A, R452A, D477A/Y478A, L498A/F500A, V509A were flown over the same mRobo3 FN1-3 channel on the SA chip, followed by a 20 s 10 mM EDTA pH 8, 150 mM NaCl injection to remove any left-over specific and non-specific binding. Duplicated titration points show that no significant surface activity (Robo3) is lost throughout the experiment.

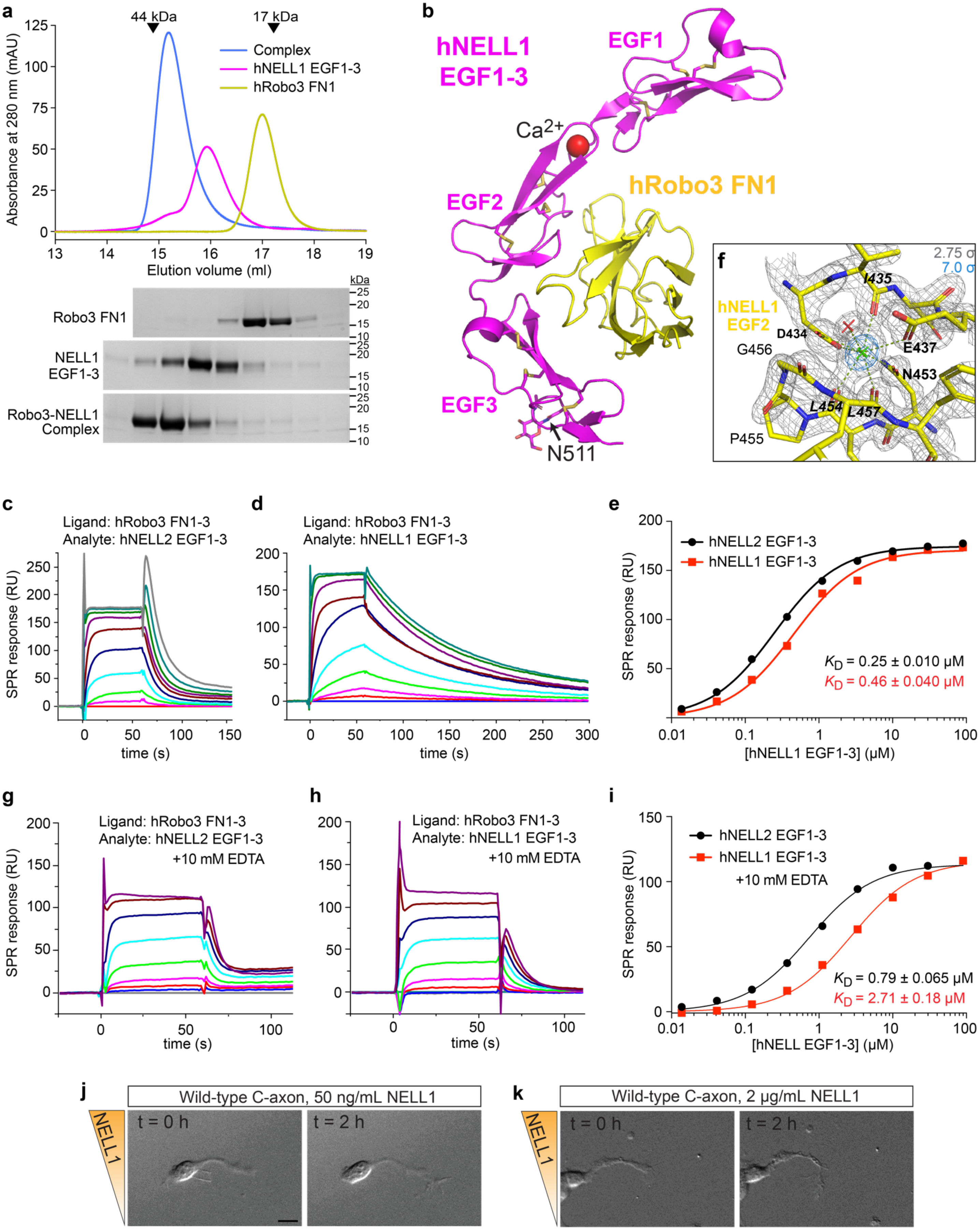
a
44 kDa
17 kDa
Complex
hNELL1 EGF1-3
hRobo3 FN1
Absorbance at 280 nm (mAU)
Elution volume (ml)
Robo3 FN1
NELL1 EGF1-3
Robo3-NELL1 Complex
kDa
b
hNELL1 EGF1-3
EGF1
Ca2+
EGF2
hRobo3 FN1
EGF3
N511
f
2.75 σ
7.0 σ
hNELL1 EGF2
I435
D434
G456
E437
N453
L454
L457
P455
c
Ligand: hRobo3 FN1-3
Analyte: hNELL2 EGF1-3
SPR response (RU)
time (s)
d
Ligand: hRobo3 FN1-3
Analyte: hNELL1 EGF1-3
time (s)
e
hNELL2 EGF1-3
hNELL1 EGF1-3
SPR response (RU)
KD = 0.25 ± 0.010 μM
KD = 0.46 ± 0.040 μM
[hNELL1 EGF1-3] (μM)
g
Ligand: hRobo3 FN1-3
Analyte: hNELL2 EGF1-3
+10 mM EDTA
SPR response (RU)
time (s)
h
Ligand: hRobo3 FN1-3
Analyte: hNELL1 EGF1-3
+10 mM EDTA
time (s)
i
hNELL2 EGF1-3
hNELL1 EGF1-3
+10 mM EDTA
SPR response (RU)
KD = 0.79 ± 0.065 μM
KD = 2.71 ± 0.18 μM
[hNELL EGF1-3] (μM)
j
Wild-type C-axon, 50 ng/mL NELL1
NELL1
t = 0 h
t = 2 h
k
Wild-type C-axon, 2 μg/mL NELL1
NELL1
t = 0 h
t = 2 h

Supplementary Fig. 4. NELL1 binds Robo3. (a) Size-exclusion chromatography of the hNELL1 EGF1-3-hRobo3 FN1 complex. hNELL1 EGF1-3, hRobo3 FN1, and a molar 1:1 mixed complex samples were injected on a Superdex 200 Increase 10/300 column, and the elution profile was recorded by following absorbance at 280 nm absorbance with an absorbance path length of 0.2 cm. Blue: hNELL2 EGF1-6 + hRobo3 FN1-3 complex sample; Yellow: hRobo3 FN1; Magenta: hNELL1 EGF1-3. SDS-PAGE for fractions from each SEC run are pasted below the chromatogram. Since the hNELL1 EGF1-3 and hRobo3 FN1 constructs are identical in size in SDS-PAGE, only a single band is observed in the complex run. **(b)** The NELL1-Robo3 structure. Red ball indicates the calcium ion bound to EGF2, and an N-linked glycan on residue N511 is depicted in sticks representation. **(c-e)** SPR sensorgrams (c and d) and binding isotherms (e) for biotinylated hRobo3 FN1-3 as ligand (coupled to SA chip), hNELL1 EGF1-3 or hNELL2 EGF1-3 as analyte (in mobile phase). Data were collected on a Biacore 3000 (GE Healthcare). hNELL1 and hNELL2 EGF1-3 have similar affinities for hRobo3 FN1-3. **(f)** Electron density for the calcium ion bound to EGF2 domain in hNELL1. This site is conserved among vertebrate and invertebrate NELL sequences (Supplementary Figure 2d). Two contour levels were used to draw electron density, where the lower cutoff shows protein density and the higher cutoff only shows the electron-rich calcium ion. Bold residue labels indicate amino acids with side chains coordinating calcium. Bold and italic labels indicate amino acids that coordinate calcium with main chain carbonyl oxygens. Additionally, two amino acids, a Proline and a Glycine, help reorient the main chain for the formation of the calcium coordination sites, and is generally conserved (Supplementary Fig. 2d) Green star: Calcium ion; Red star: Ca^{2+}-coordinated water molecule. **(g-i)** Repeat of panels c-e, but with additional 10 mM EDTA in all buffers. EDTA causes mild loss of Robo3 affinity for NELL1 and NELL2. **(j,k)** DIC images of commissural axons exposed to 50 ng/ml (j) or 2 µg/ml (k) of NELL1 (0 h and 2 h). Commissural axons are repelled by 2 µg/ml, but not 50 ng/ml NELL1. Scale bar, 10 µm (j, k).

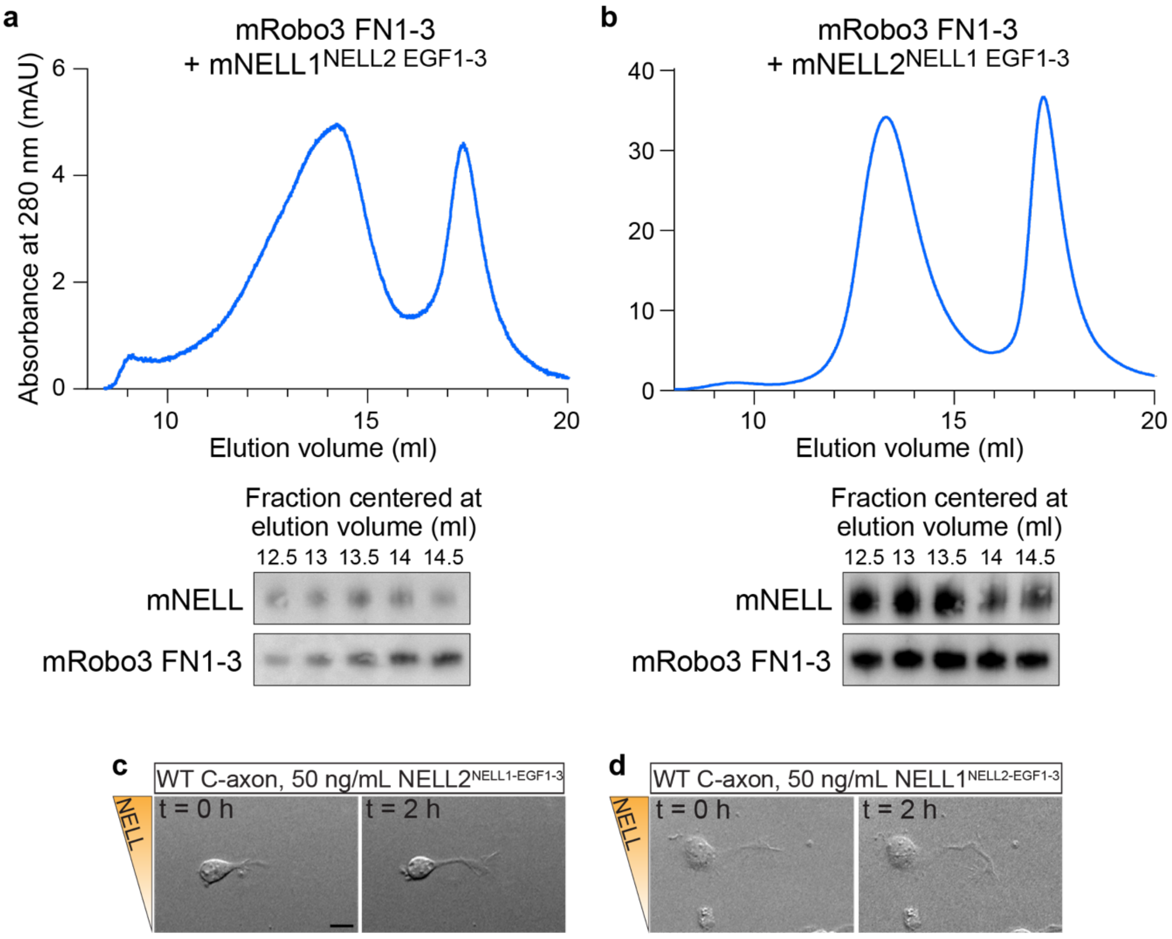

Supplementary Fig. 5. Chimeras of NELLs can bind Robo3. (a,b) mNELL1 and mNELL2 with their EGF1-3 domains swapped can still bind mRobo3 FN1-3 as they co-elute on a Superose 6 10/300 SEC column. The presence of both proteins in the complex peak was confirmed via western blotting with an anti-His antibody. **(c,d)** DIC images of commissural axons exposed to 50 ng/ml of NELL EGF1-3 domain swaps (0 h and 2 h). Commissural axons are only repelled by 50 ng/ml NELL1$^{NELL2\text{-}EGF1\text{-}3}$. Scale bar, 10 µm (c, d).

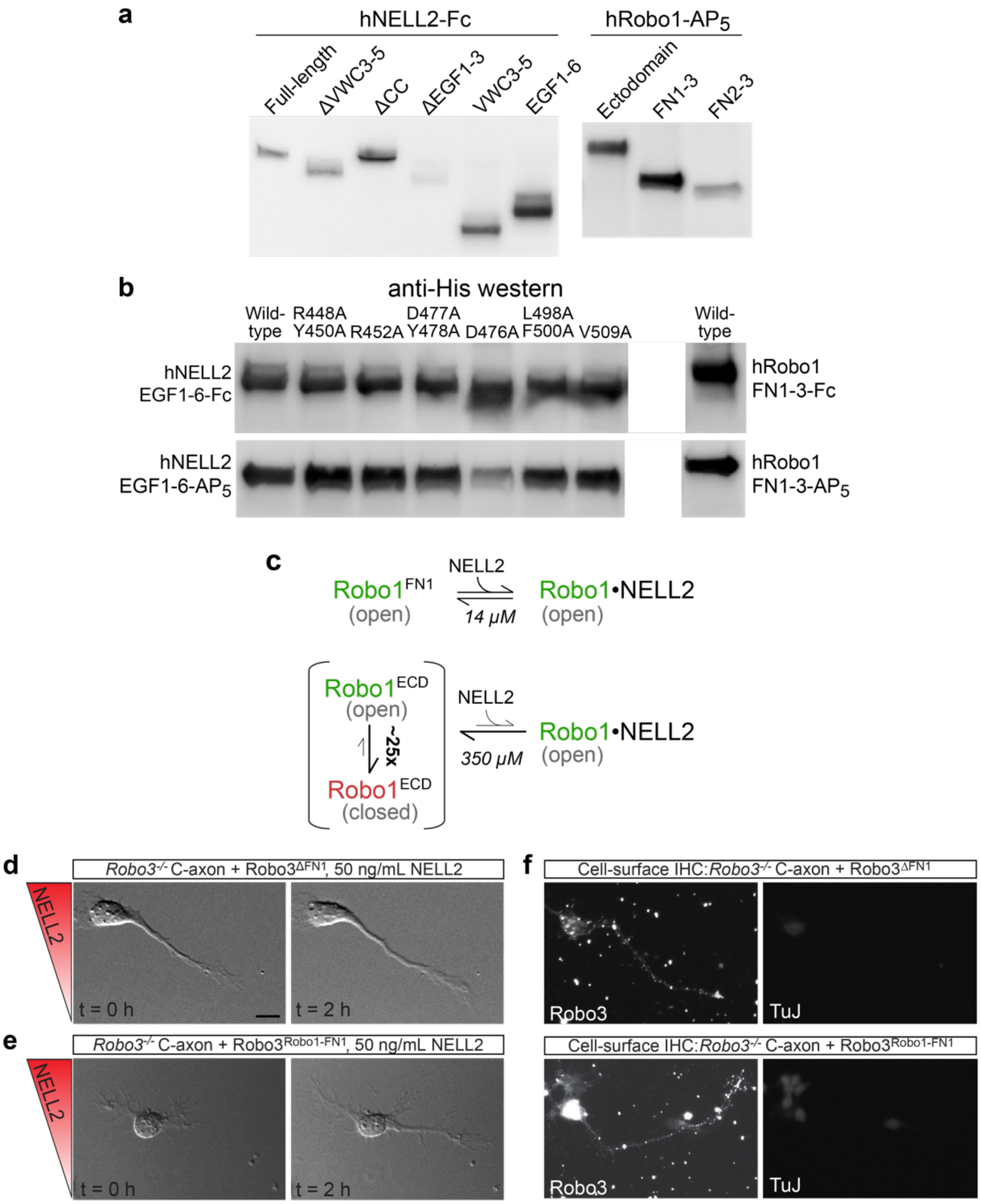

Supplementary Fig. 6. Robo1 has weak affinity for NELL2, and its FN1 domain cannot mediate NELL2-dependent axon repulsion in the context of Robo3.1. (a) Western blot for testing expression levels of constructs used on Figure 6c. **(b)** Western blot used for normalizing expression levels of hRobo1 and hNELL1 mutants used in the ECIA experiment in Figure 6d. **(c)** A thermodynamic model to explain the affinities of NELL2 for Robo1 ECD vs. FN1 domain. The values in italics are dissociation constants. Robo1 closed state is favored over open state by ~25 fold. Robo1 closed state represents either the full-ectodomain model provided by Aleksandrova (2018) or by Barak (2019), in both of which the NELL-binding site is blocked. Robo1 FN1 domain serves as a proxy in our experiments for the open state in terms of NELL binding. **(d,e)** DIC images of *Robo3*$^{-/-}$ commissural axons expressing Robo3$^{\Delta FN1}$ (d) or Robo3$^{Robo1\text{-}FN1}$ (e) exposed to NELL2 (0 h and 2 h). Neither mutant form of Robo3 restores axon turning in response to NELL2. **(f).** Non-permeabilized immunolabeling with Robo3 ectodomain antibody demonstrates Robo3$^{\Delta FN1}$ (top) or Robo3$^{Robo1\text{-}FN1}$ (bottom) expression on the surface of *Robo3*$^{-/-}$ axons after electroporation. Scale bar, 10 µm (d-f).

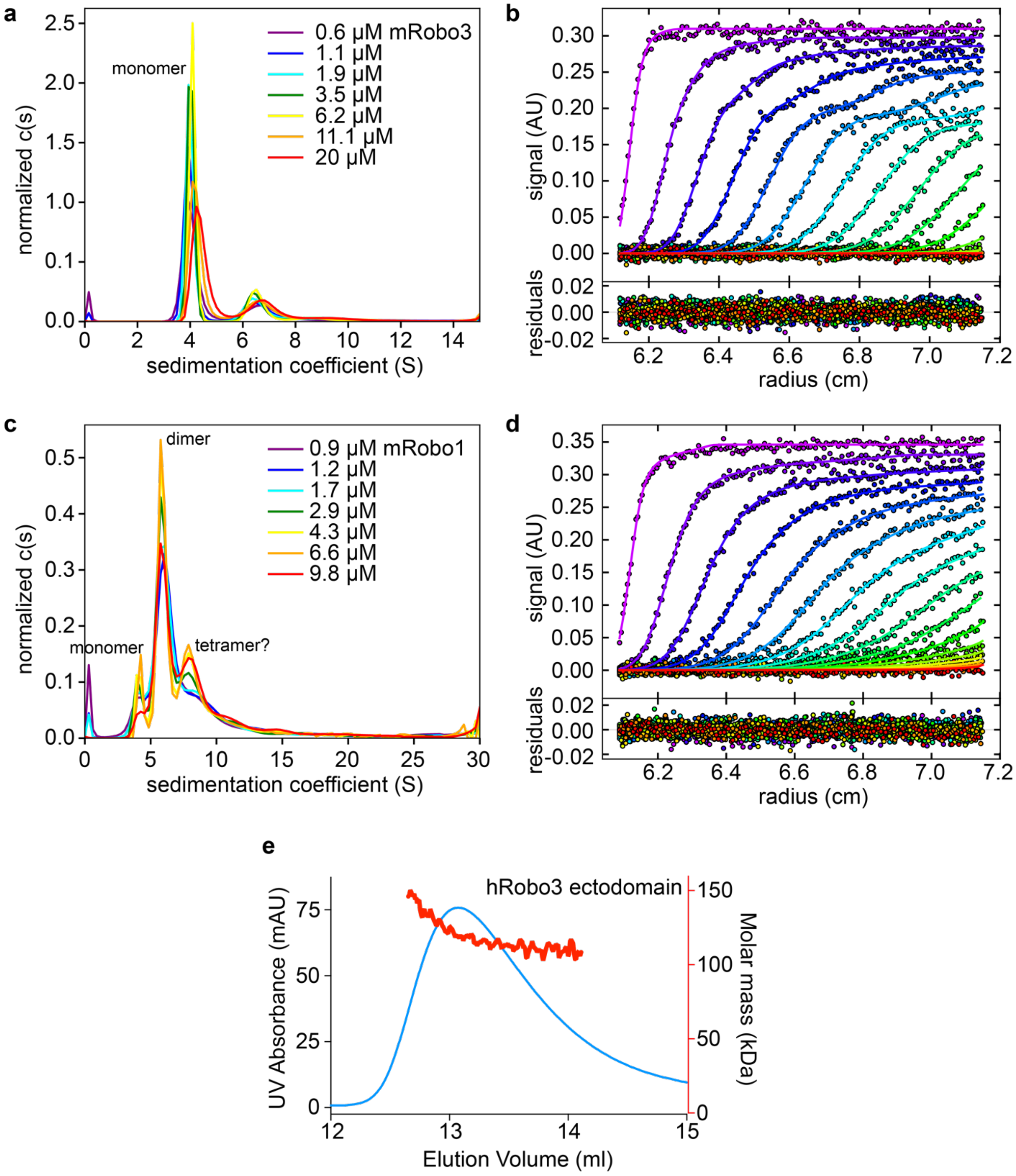

Supplementary Fig. 7. Oligomeric states of Robo1 and Robo3 as measured by AUC and MALS. (a,b) mRobo3 is monomeric, as determined by sedimentation velocity analytical ultracentrifugation (AUC). The height of the minor peak at 6.5 S does not change with increasing concentrations of Robo3, indicating that the peak might represent a contaminant or a fraction of protein, possibly misfolded, and not in equilibrium with the monomer. **(c,d)** mRobo1 exists primarily as a dimer, but in equilibrium with monomer and higher-order oligomers. With increasing concentrations, the population moves towards higher oligomeric states, indicative of an exchange between the oligomeric states. **(e)** Molar mass measurements using MALS for hRobo3 ectodomain. Predicted monomeric molecular weight for hRobo3 is 108 kDa, accounting for likely N-linked glycans. Average molar mass measured for hRobo3 is 117 kDa, strongly indicative of a monomer. UV absorbance data was collected on an ÄKTA FPLC with a UV pathlength of 1 cm.

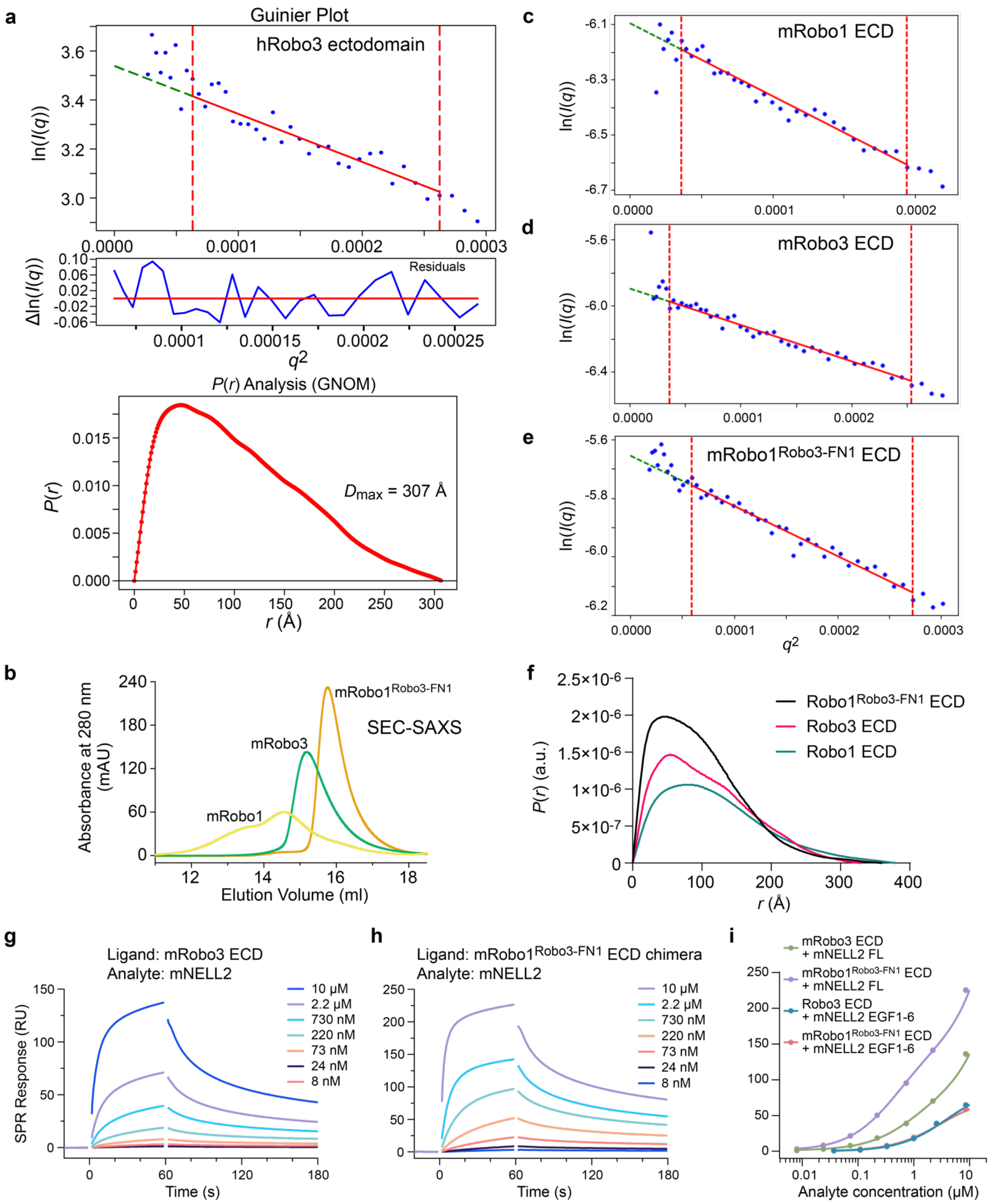

j

	mRobo1	mRobo3	mRobo1$^{Robo3\text{-}FN1}$
K_D (mNELL2)	>100 μM	0.94 μM	0.47 μM
R_g	89 Å	81 Å	71 Å

Supplementary Fig. 8. Robo1$^{Robo3\text{-}FN1}$ chimera has an elongated shape, and strongly binds NELL2 . (a) Linear Guinier plots for hRobo3 ectodomain collected at APS BioCAT SAXS beamline. Plots drawn in *BioXTAS RAW* version 1.5.1. Robo3 has a highly elongated shape as seen in the $P(r)$ plot and a D_{max} of 307 Å roughly corresponding with eight IG/FNIII domains (~4 Å) strung together end-to-end, as observed in the *DAMMIF* bead models (**Fig. 7c**). Predicted molecular weight matches that of a Robo3 ectodomain monomer. (**b**) SEC-SAXS runs show that the mRobo1Robo3-FN1 chimera is monomeric. The higher R_g values reflect mixed oligomeric states. **(c-f)** Linear Guinier plots used to measure R_g (c-e) and $P(r)$ analysis (f) for Robo1 ECD, Robo3 ECD and Robo1$^{Robo3\text{-}FN1}$ chimera, performed using *GNOM* within *BioXTAS RAW* version 1.6.3. **(g-i)** SPR analysis for binding of mNELL2 to Robo3 and Robo1$^{Robo3\text{-}FN1}$ chimera. Equilibrium binding isotherms are plotted in (i) and fit to a 1:1 binding model with a non-specific binding term. **(j)** Summary table for mNELL2 affinity and radius of gyration measurements for mRobo1, mRobo3 and the Robo1$^{Robo3\text{-}FN1}$ chimera.

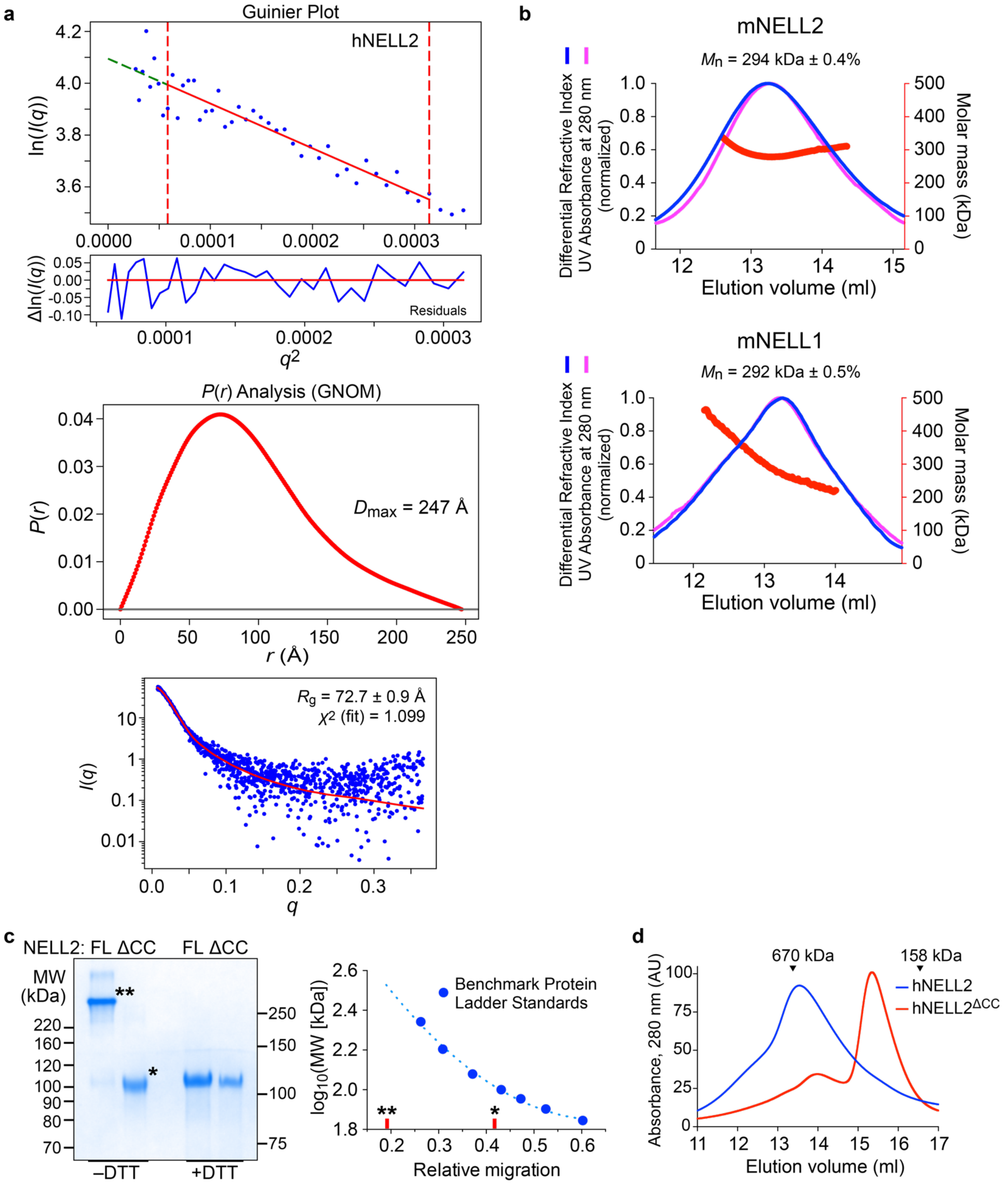

Supplementary Fig. 9. Oligomeric states of NELL1 and NELL2. (a) (Top Panel) Linear Guinier plot for hNELL2 sample collected at APS BioCAT SAXS beamline. Plots drawn in *BioXTAS RAW* version 1.5.1.

(middle panel) $P(r)$ analysis, performed using *GNOM* within *BioXTAS RAW* (Fig. 7e). NELL2 has a more compact shape than Robo ECDs, but is still a non-globular trimer, as predicted by the molecular weight analysis. **(b)** Molar mass measurements using MALS for mNELL2 and mNELL1. Predicted monomeric molecular weights for mNELL2 and mNELL1 accounting for likely N-linked glycans, are 99 kDa and 101 kDa, respectively. For molar mass estimation, dn/dc values of 0.181 and 0.179, based on predicted N-linked glycan compositions, were used for mNELL2 and mNELL1, respectively. Measured molar mass values are indicative of trimerization. **(c)** NELL2 forms trimers via disulfide linkages at or near its CC domain. NELL2 and NELL2 deletion of the CC domain and four cysteines (NELL2$^{\Delta CC}$) are run on non-reducing (–DTT) and reducing (+DTT) SDS-PAGE. * indicates monomeric NELL2$^{\Delta CC}$; ** indicates full-length NELL2 in non-reducing lanes. (Right) Molecular weight predictions based on protein ladder standards. **(d)** Size-exclusion chromatography of hNELL2 and hNELL2$^{\Delta CC}$. hNELL2 and hNELL2$^{\Delta CC}$ were injected separately on a Superose 6 Increase 10/300 column, and the elution profiles were recorded by following absorbance at 280 nm with an absorbance path length of 0.2 cm. Blue: hNELL2, Red: hNELL2$^{\Delta CC}$. Elution volumes for protein size standards are depicted as triangles above the chromatograms. Both NELL2 and NELL2$^{\Delta CC}$ run on SEC at sizes about 1.5 to 2-fold predicted from their oligomeric state. This is likely a result of their non-globular shapes.

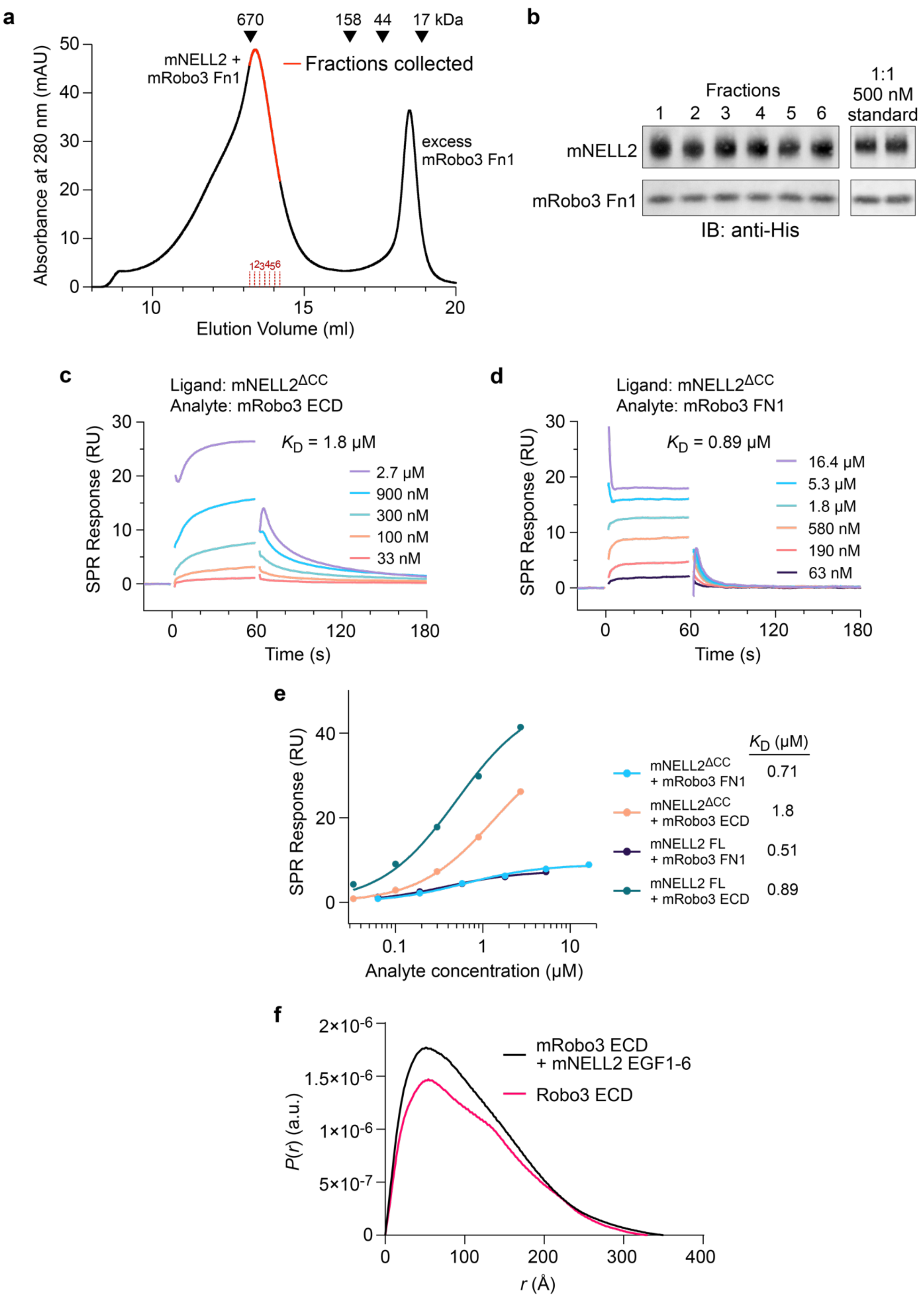

Supplementary Fig. 10. The coiled-coil domain of NELL2 does not contribute significantly to binding Robo3. (a-b) mNELL2 and mRobo3 FN1 form a complex that migrates as a large oligomer in SEC, and incompatible with a simple 1+1 complex. Quantitation of NELL2:Robo3 using a western blot (b) shows an

equimolar complex. The samples are run on a Superose 6 10/300 column and the path length of the UV detector is 0.2 cm. The estimated concentrations across the complex fractions yielded a 1.16:1 mNELL2:mRobo3 FN1 molar ratio. **(c-e)** Surface plasmon resonance experiments for full-length NELL2 and NELL2$^{\Delta CC}$ against the mRobo3 ECD and FN1 domain. The raw chromatograms for NELL2$^{\Delta CC}$ to mRobo3 ECD and to FN1 domain are shown in (c) and (d), respectively. The binding isotherms for the interaction data and the dissociation constants are in (e).

Supplementary Table 1. Data and refinement statistics for x-ray crystallography.

	**hRobo3 FNIII 1 + hNELL2 EGF 1-6**	**hRobo3 FNIII 2-3**	**hRobo3 FNIII 1 + hNELL1 EGF 1-3**
**Data Collection**			
Space Group	$P2_12_12_1$	$P2_1$	$P3_121$
Cell Dimensions			
a, *b*, *c* (Å)	41.87, 90.44, 171.58	54.99, 36.61, 64.90	87.00, 87.00, 211.30
α, β, γ (°)	90, 90, 90	90, 114.44, 90	90, 90, 120
Resolution (Å)	50-2.76 (2.92-2.76)*	55-1.80 (1.91-1.80)	55-1.80 (1.85-1.80)
R_{sym} (%)	10.6 (159.9)	6.6 (86.0)	23.8 (205.3)
$\langle I \rangle / \langle \sigma(I) \rangle$	10.53 (0.81)	6.59 (0.79)	5.75 (0.72)
$CC_{1/2}$	99.8 (43.0)	99.6 (58.6)	99.6 (46.5)
Completeness (%)	99.4 (96.9)	99.7 (98.1)	99.6 (95.9)
Redundancy	6.4 (6.5)	1.8 (1.6)	13.0 (6.2)
**Refinement**			
Resolution (Å)	50-2.76 (2.87-2.76)*	50.1-1.80 (1.88-1.80)	51.45-1.80 (1.85-1.80)
Reflections	17,498	21,770	85,786
R_{cryst} (%)	23.68 (35.27)	21.73 (42.14)	22.99 (53.83)
R_{free} (%)***	26.37 (36.00)	24.31 (41.09)	26.64 (52.77)
Number of atoms			
Protein	2392	1474	5141
Ligand/Glycans	55	6	113
Water	N/A	138	642
Average B-factors ($Å^2$)			
All	122.7	48.8	45.3
Protein	122.1	49.2	44.5
Ligand/Glycans	150.0	73.5	64.3
Solvent	N/A	43.8	48.2
R.m.s. deviations from ideality			
Bond Lengths (Å)	0.003	0.002	0.007
Bond Angles (°)	0.594	0.531	0.867
Ramachandran statistics			
Favored (%)	89.06	97.92	96.46
Outliers (%)	0.30	0	0
Rotamer Outliers (%)	3.57	0.63	0.35
All-atom Clashscore ‡	3.75	3.37	1.79
Coordinate Error § (Å)	0.44	0.28	0.30

* The values in parentheses are for reflections in the highest resolution bin.

** 7.5% (1315), 5.0 (1083) and 2.3% (1985) of reflections were not used during refinement for cross validation.

Supplementary Table 2. Summary of SPR results in this study.

Ligand (stationary phase)	Analyte (mobile phase)	Kd (µM)	S.E. of the fit
hRobo3 FN1-3	hNELL2 EGF1-6	0.579	0.043
mRobo3 FN1-3	mNELL2 EGF1-6 WT	0.710	0.032
	mNELL2 EGF1-6 R448A/Y540A	87	6.6
	mNELL2 EGF1-6 R452A	1.7	0.085
	mNELL2 EGF1-6 D477A/Y478A	≥100	-
	mNELL2 EGF1-6 L498A/F500A	≥100	-
	mNELL2 EGF1-6 V509A	1.7	0.064
mRobo1 ECD	mNELL2	~350	-
mRobo1 FN1-3	mNELL2 EGF1-6	13.8	0.34
hRobo3 FN1-3	hNELL2 EGF1-3	0.25	0.010
	hNELL2 EGF1-3 + 10 mM EDTA	0.79	0.065
	hNELL1 EGF1-3	0.46	0.040
	hNELL1 EGF1-3 + 10 mM EDTA	2.71	0.18
mRobo3 ECD	mNELL2	0.94	0.15
	mNELL2 EGF1-6	1.7	0.21
mRobo1$^{Robo3\text{-}FN1}$ ECD	mNELL2	0.47	0.024
	mNELL2 EGF1-6	1.7	0.19
mNELL2	mRobo3 ECD	0.89	0.14
	mRobo3 FN1	0.51	0.071
mNELL2$^{\Delta CC}$	mRobo3 ECD	1.8	0.16
	mRobo3 FN1	0.72	0.13

Supplementary Table 3. SAXS analysis performed in this study.

	hRobo3 ECD	hNELL2	mRobo1 ECD	mRobo3 ECD	mRobo1 Robo3-FN1	mRobo3 ECD + mNELL2 EGF1-6
**Data-collection parameters**						
Instrument	Advanced Photon Source at the Argonne National Laboratory, BioCAT 18-ID beamline with Pilatus 3X 1M detector					
Date of data collection	2018-06-30	2018-06-30	2019-11-08	2019-11-08	2019-11-08	2019-11-08
Beam size (µm)	172 x 172					
q range (Å^{-1})	0.004 – 0.36					
Exposure time	0.5 second images					
Concentration (mg ml^{-1})	~2					
Temperature (K)	295					
**Structural parameters**						
$I(0)$ (cm^{-1}) [from $P(r)$]	36.0 ± 0.7	59.0 ± 0.8	0.0023 ± 0.000022	0.0028 ± 0. 000023	0.0036 ± 0.000035	0.0034 ± 0.000019
R_g (Å) [from $P(r)$]	86.3 ± 1.8	72.7 ± 0.9	98.9 ± 1.4	87.8 ± 1.1	80.4 ± 1.7	85.7 ± 0.6
$I(0)$ (cm^{-1}) (from Guinier)	34.4 ± 1.2	60.0 ± 1.3	0.0023 ± 0.000039	0.0028 ± 0.000023	0.0035 ± 0.000041	0.0033 ± 0.000021
R_g (Å) (from Guinier)	76.7 ± 3.3	72.0 ± 2.0	88.9 ± 3.3	81.1 ± 1.0	71.7 ± 2.4	78.2 ± 0.8
D_{max} (Å)	310	250	380	330	360	310
Porod volume estimate (Å^3)	323000	573000	469000	304000	269000	378000
Dry volume calculated from sequence (Å^3)*	115000	334000 (trimer)	218000 (dimer)	115000	112000	151000

* N-linked glycans attached to Robos and NELLs are ignored.

Supplementary Table 4. Primer sequences used for cloning.

(a) Cloning into pAcGP67A

Species	Gene name	For/Rev	Primer sequence (5' – 3')
mouse	NELL1 FL	F	CTGCCTTTGCGGCGGATCCCGACCCTGACCTTCAGATGGACATCATC
mouse	NELL1 FL	R	GATGGTGGTGATGGTGGAATTCATTCTCAAGACACACCAGATCCACAG
mouse	NELL2 FL	F	CTGCCTTTGCGGCGGATCCCGGTGTGGACCCCTCCCTACAG
mouse	NELL2 FL	R	GATGGTGGTGATGGTGGAATTCCAGCTCCTGAAGGCACTGTGG
human	NELL1 FL	F	CTGCCTTTGCGGCGGATCCCTTTGGGATGGACCCTGACCTTCAG
human	NELL1 FL	R	GATGGTGGTGATGGTGGAATTCATTATTTGAAGACACTCAAAATCCACAGAACAAC
human	NELL2 FL	F	CTGCCTTTGCGGCGGGTTATGACTTTTGTTCTG
human	NELL2 FL	R	CGGCCGCTCCGGTTAGTGGTGATGGTGATGATGCAGTTCCTGAAGGCACTGTG
mouse	Robo1 ECD	F	CTGCCTTTGCGGCGGATCCCCGCATTGTTGAACACCCTTCAGAC
mouse	Robo1 ECD	R	GATGGTGGTGATGGTGGAATTCAGACACGGGGTTTCCGTG
mouse	Robo3 ECD	F	CTGCCTTTGCGGCGGATCCCAGGGACATCTCCAACTCCAGCG
mouse	Robo3 ECD	R	GATGGTGGTGATGGTGGAATTCCTTCCGCAGCACCTTAGCCAAC
human	Robo1 ECD	F	CTGCCTTTGCGGCGGATCCCCGCATTGTTGAACACCCTTCAG
human	Robo1 ECD	R	GATGGTGGTGATGGTGGAATTCGAGGATGATCCAACAGGCTGC
human	Robo3 ECD	F	CTGCCTTTGCGGCGGGGGACATCTCCAACTCCAG
human	Robo3 ECD	R	CGGCCGCTCCGGTTAGTGGTGATGGTGATGATGGGGCTCCCGCAGCAC
human	NELL2 EGF1-6	F	CTGCCTTTGCGGCGGGTTATGACTTTTGTTCTG
human	NELL2 EGF1-6	R	CGGCCGCTCCGGTTAGTGGTGATGGTGATGATGCCCTGTGCAATTCTTTCCATG
human	Robo3 FN1-3	F	CTGCCTTTGCGGCGGAACCCAGTTCCCCTCC
human	Robo3 FN1-3	R	CGGCCGCTCCGGTTAGTGGTGATGGTGATGATGCAGGTCCGGCGGGG

(b) Cloning into pECIA-bait and pECIA-prey

Species	Gene name	For/Rev	Primer sequence (5' – 3')
human	NELL2 EGF1-6	F	CTCGCTCGGGGAGGTACCTGGTTATGACTTTTGTTCTGAAAGGCATAAC
human	NELL2 EGF1-6	R	GTGCTGGATATCTGCAGAATTTGTGCAATTCTTTCCATGAGGACATCGAC
human	Robo1 FN1-3	F	CTCGCTCGGGGAGGTACCTAATTTAATCCCTAGTGCCCCATCAAAAC
human	Robo1 FN1-3	R	GTGCTGGATATCTGCAGAATTGAGGATGATCCAACAGGCTGC
human	Robo1 ECD	F	CTCGCTCGGGGAGGTACCTCGCATTGTTGAACACCCTTCAG
human	Robo1 ECD	R	GTGCTGGATATCTGCAGAATTGAGGATGATCCAACAGGCTGC
human	Robo1 FN2-3	F	CTCGCTCGGGGAGGTACCTGAAGTGCACTGGACAGTAGATCAACAG
human	Robo1 FN2-3	R	GTGCTGGATATCTGCAGAATTGAGGATGATCCAACAGGCTGC
human	NELL2 FL	F	CTCGCTCGGGGAGGTACCTCTTGGTGTGGACCCTTCCCTAC
human	NELL2 FL	R	GTGCTGGATATCTGCAGAATTCAGTTCCTGAAGGCACTGTGGATC
human	NELL2 ΔVWC3-5	R	GTGCTGGATATCTGCAGAATTTGTGCAATTCTTTCCATGAGGACATCGAC
human	NELL2 VWC3-5	F	CTCGCTCGGGGAGGTACCTGGGGACTGCATCCATGATGGAAAAG
human	NELL2 VWC3-5	R	GTGCTGGATATCTGCAGAATTCAGTTCCTGAAGGCACTGTGGATC

(c) Mutagenesis primers

Species	Mutation name	For/Rev	Primer sequence (5' – 3')
human	NELL2 R448A-Y450A	F	CGATGAGTGTGCTGAAGGGGCCCATGCCTGTCGTGAAAATACAATG
human	NELL2 R448A-Y450A	R	CATTGTATTTTCACGACAGGCATGGGCCCCTTCAGCACACTCATCG
human	NELL2 R452A	F	GGGCGCCATTACTGTGCCGAAAATACAATGTGTGTC
human	NELL2 R452A	R	GACACACATTGTATTTTCGGCACAGTAATGGCGCCC
human	NELL2 D477A-Y478A	F	CATCAGAATTGATGCCGCCTCATGTACAGAACATGATGAGTG
human	NELL2 D477A-Y478A	R	CATCATGTTCTGTACATGAGGCGGCATCAATTCTGATGTATCC
human	NELL2 D476A	F	GGATACATCAGAATTGCCGATTATTCATGTACAGAACATG
human	NELL2 D476A	R	CATGAATAATCGGCAATTCTGATGTATCCAGTTTTGCAG
human	NELL2 L498A-F500A	F	CTGTGATGAAAATGCTGCCTGCGCCAACACTGTTGGAGGACAC
human	NELL2 L498A-F500A	R	GTGTCCTCCAACAGTGTTGGCGCAGGCAGCATTTTCATCACAG
human	NELL2 V509A	F	GGACACAACTGTGCCTGCAAGCCGGGC
human	NELL2 V509A	R	GCCCGGCTTGCAGGCACAGTTGTGTCC

human	Robo3 T604A	F	TGGCGTGCCGTGGCAGATGGCGTGC
human	Robo3 T604A	R	CCATCTGCCACGGCACGCCATGTGTTG
human	Robo3 E639A	F	GCCTCAGTGCCCCCAGCCCCGTCTC
human	Robo3 E639A	R	GACGGGGCTGGGGGCACTGAGGCCC
human	Robo3 R630A	F	CCTGTTTCTGGTTGCCGCAGTGGGAGCCTGGG
human	Robo3 R630A	R	CCAGGCTCCCACTGCGGCAACCAGAAACAGGTAG
human	Robo3 W635A	F	GCAGTGGGAGCCGCCGGCCTCAGTGAGCC
human	Robo3 W635A	R	GCTCACTGAGGCCGGCGGCTCCCACTGC
human	Robo3 N600A-T601A	F	CCCAGCAGCTGGCGCCGCCTGGCGTACTGTGG
human	Robo3 N600A-T601A	R	CCACAGTACGCCAGGCGGCGCCAGCTGCTGGG
human	Robo3 F594A-W602A	F	GCCCAGCAGCTGGCAACACAGCCCGTACTGTGGC
human	Robo3 F594A-W602A	R	GGGCTGTGTTGCCAGCTGCTGGGCTGGCGGCCTCTATCAC
human	Robo3 I624A-L626A	F	GCCCAATACCGCCTACGCCTTTCTGGTTCGAGCAGTGGGAGC
human	Robo3 I624A-L626A	R	GCTCGAACCAGAAAGGCGTAGGCGGTATTGGGCTGCAGACCG
mouse	NELL2 R448A-Y450A	F	GCGCCATTACTGCGCCGAGAACACCATGTGTGTG
mouse	NELL2 R448A-Y450A	R	CACACACATGGTGTTCTCGGCGCAGTAATGGCGCC
mouse	NELL2 R452A	F	GCGCCATTACTGCGCCGAGAACACCATGTGTGTG
mouse	NELL2 R452A	R	CACACACATGGTGTTCTCGGCGCAGTAATGGCGCC
mouse	NELL2 D477A-Y478A	F	ACATCAGGATCGACGCCGCCTCATGTACAGAACATGATGAG
mouse	NELL2 D477A-Y478A	R	CATGTTCTGTACATGAGGCGGCGTCGATCCTGATGTACCC
mouse	NELL2 L498A-F500A	F	GAAAACGCTGCCTGCGCCAACACTGTTGGAGGACACAACTGTGTC
mouse	NELL2 L498A-F500A	R	GTCCTCCAACAGTGTTGGCGCAGGCAGCGTTTTCATCACAATTGTGC
mouse	NELL2 V509A	F	CACAACTGTGCCTGCAAGCCTGGC
mouse	NELL2 V509A	R	CCAGGCTTGCAGGCACAGTTGTGTC

Table S5. Table of plasmids generated and used in this study.

We use the initial methionine starting M^{1}ESRV… for the mouse NELL2 gene, matching the annotated human NELL2 N terminus. FL: full length, ECD: extracellular domains or ectodomain, WT: wild type, Δ: deletion.

(a) Plasmids used in AP-staining experiments and Dunn chamber turning assays, Fig. 1g, 2a, b and 6g, h.

Construct	Promoter	Species	Amino acids	Fusion Protein
NELL2$^{\text{EGF1-3}}$	CMV	Human	G397-K522	C-terminal AP
NELL2$^{\text{EGF4-6}}$	CMV	Human	A523-T637	C-terminal AP
NELL2$^{\text{EGF1-2}}$	CMV	Human	G397-T481	C-terminal AP
NELL2$^{\text{EGF2}}$	CMV	Human	D440-T481	C-terminal AP
NELL2$^{\text{EGF3}}$	CMV	Human	E482-K522	C-terminal AP
NELL2$^{\text{EGF2-3}}$	CMV	Human	D440-K522	C-terminal AP
Robo3$^{\text{FN1}}$	CMV	Human	M1-D72, P555-V658, P865-R1386	None
Robo3$^{\Delta\text{IG1-FN1}}$	CMV	Human	M1-D72, Q655-R1386	None
Robo3$^{\Delta\text{FN1}}$	CAG	Mouse	M1-D541, S653-R1402	None
Robo3$^{\text{Robo1-FN1}}$	CAG	Mouse	M1-A544, Robo1 P512-D617, A658-R1402	None
Robo3^{FL}	CMV	Human	M1-R1386	None
Robo3.1	CAG	Mouse	M1-R1402	None
Robo3.2	CAG	Mouse	M1-K1345	None

(b) Constructs for protein binding via cell-staining, expressed in S2 or High Five cells, Fig. 3d, e.

Construct	Promoter	Species	Amino acids	Fusion Protein
NELL2$^{\text{EGF1-6 WT}}$	Actin 5C	Human	G397- T637	C-terminal FLAG
NELL2$^{\text{EGF1-6 R448A-Y450A}}$	Actin 5C	Human	G397- T637	C-terminal FLAG
NELL2$^{\text{EGF1-6 R452A}}$	Actin 5C	Human	G397- T637	C-terminal FLAG
NELL2$^{\text{EGF1-6 D477A-Y478A}}$	Actin 5C	Human	G397- T637	C-terminal FLAG
NELL2$^{\text{EGF1-6 D476A}}$	Actin 5C	Human	G397- T637	C-terminal FLAG
NELL2$^{\text{EGF1-6 L498A-F500A}}$	Actin 5C	Human	G397- T637	C-terminal FLAG
NELL2$^{\text{EGF1-6 V509A}}$	Actin 5C	Human	G397- T637	C-terminal FLAG
Robo3$^{\text{FN1-3 WT}}$	Actin 5C	Human	P549- L870	C-terminal FLAG
Robo3$^{\text{FN1-3 T604A}}$	Actin 5C	Human	P549- L870	C-terminal FLAG
Robo3$^{\text{FN1-3 E639A}}$	Actin 5C	Human	P549- L870	C-terminal FLAG
Robo3$^{\text{FN1-3 R630A}}$	Actin 5C	Human	P549- L870	C-terminal FLAG
Robo3$^{\text{FN1-3 W635A}}$	Actin 5C	Human	P549- L870	C-terminal FLAG
Robo3$^{\text{FN1-3 N600A-T601A}}$	Actin 5C	Human	P549- L870	C-terminal FLAG
Robo3$^{\text{FN1-3 F594A-W602A}}$	Actin 5C	Human	P549- L870	C-terminal FLAG
Robo3$^{\text{FN1-3 I624A-L626A}}$	Actin 5C	Human	P549- L870	C-terminal FLAG
NELL2$^{\text{EGF1-6 WT}}$	Polyhedrin	Human	G397- T637	C-terminal Avi and His tags
Robo3$^{\text{FN1-3 WT}}$	Polyhedrin	Human	P549- L870	C-terminal Avi and His tags

(c) ECIA constructs used in Fig. 6c, d and Supplemental Fig. 3c.

Construct	Promoter	Species	Amino acids	Fusion Protein
NELL2$^{\text{EGF1-6 WT}}$	Actin 5C	Human	G397- T637	C-terminal Fc
NELL2$^{\text{EGF1-6 R448A-Y450A}}$	Actin 5C	Human	G397- T637	C-terminal Fc
NELL2$^{\text{EGF1-6 R452A}}$	Actin 5C	Human	G397- T637	C-terminal Fc
NELL2$^{\text{EGF1-6 D477A-Y478A}}$	Actin 5C	Human	G397- T637	C-terminal Fc
NELL2$^{\text{EGF1-6 D476A}}$	Actin 5C	Human	G397- T637	C-terminal Fc
NELL2$^{\text{EGF1-6 L498A-F500A}}$	Actin 5C	Human	G397- T637	C-terminal Fc
NELL2$^{\text{EGF1-6 V509A}}$	Actin 5C	Human	G397- T637	C-terminal Fc
NELL2$^{\text{EGF1-6 WT}}$	Actin 5C	Human	G397- T637	C-terminal AP
NELL2$^{\text{EGF1-6 R448A-Y450A}}$	Actin 5C	Human	G397- T637	C-terminal AP
NELL2$^{\text{EGF1-6 R452A}}$	Actin 5C	Human	G397- T637	C-terminal AP
NELL2$^{\text{EGF1-6 D477A-Y478A}}$	Actin 5C	Human	G397- T637	C-terminal AP
NELL2$^{\text{EGF1-6 D476A}}$	Actin 5C	Human	G397- T637	C-terminal AP
NELL2$^{\text{EGF1-6 L498A-F500A}}$	Actin 5C	Human	G397- T637	C-terminal AP
NELL2$^{\text{EGF1-6 V509A}}$	Actin 5C	Human	G397- T637	C-terminal AP
Robo3$^{\text{FN1-3 WT}}$	Actin 5C	Human	P549- L870	C-terminal AP

Robo3$^{\text{FN1-3 T604A}}$	Actin 5C	Human	P549- L870	C-terminal AP
Robo3$^{\text{FN1-3 E639A}}$	Actin 5C	Human	P549- L870	C-terminal AP
Robo3$^{\text{FN1-3 R630A}}$	Actin 5C	Human	P549- L870	C-terminal AP
Robo3$^{\text{FN1-3 W635A}}$	Actin 5C	Human	P549- L870	C-terminal AP
Robo3$^{\text{FN1-3 N600A-T601A}}$	Actin 5C	Human	P549- L870	C-terminal AP
Robo3$^{\text{FN1-3 F594A-W602A}}$	Actin 5C	Human	P549- L870	C-terminal AP
Robo3$^{\text{FN1-3 I624A-L626A}}$	Actin 5C	Human	P549- L870	C-terminal AP
NELL2^{FL}	Actin 5C	Human	L22-L816	C-terminal Fc
NELL2$^{\Delta\text{VWC3-5}}$	Actin 5C	Human	L22-T637	C-terminal Fc
NELL2$^{\Delta\text{CC}}$	Actin 5C	Human	L22-G236, E271-L816	C-terminal Fc
NELL2$^{\Delta\text{EGF1-3}}$	Actin 5C	Human	L22-K396, A523-L816	C-terminal Fc
NELL2$^{\text{VWC3-5}}$	Actin 5C	Human	G638-L816	C-terminal Fc
Robo1^{ECD}	Actin 5C	Human	R30-L875	C-terminal AP
Robo1$^{\text{FN1-3}}$	Actin 5C	Human	N522-L875	C-terminal AP
Robo1$^{\text{FN2-3}}$	Actin 5C	Human	E656-L875	C-terminal AP
Robo1$^{\text{FN1-3}}$	Actin 5C	Human	N522-L875	C-terminal Fc

(d) Constructs used for protein biochemistry and structural biology:

Construct	Promoter	Species	Amino acids	Fusion Protein
NELL2^{FL}	Polyhedrin	Mouse	G23-L816	C-terminal His
NELL2$^{\Delta\text{CC}}$	Polyhedrin	Mouse	G23-G236, E271-L816	C-terminal His
NELL1^{FL}	Polyhedrin	Mouse	D25-N809	C-terminal His
NELL2$^{\text{FL R448A-Y450A}}$	Polyhedrin	Mouse	G23-L816	C-terminal His
NELL2$^{\text{FL R452A}}$	Polyhedrin	Mouse	G23-L816	C-terminal His
NELL2$^{\text{FL L498A-F500A}}$	Polyhedrin	Mouse	G23-L816	C-terminal His
NELL2$^{\text{FL V509A}}$	Polyhedrin	Mouse	G23-L816	C-terminal His
NELL2$^{\text{FL R448A-Y450A}}$	Polyhedrin	Mouse	G23-L816	C-terminal His
NELL2$^{\text{FL R452A}}$	Polyhedrin	Mouse	G23-L816	C-terminal His
NELL2$^{\text{EGF1-6}}$	Polyhedrin	Mouse	G397-T637	C-terminal His
NELL2$^{\text{EGF1-6 R448A-Y450A}}$	Polyhedrin	Mouse	G397-T637	C-terminal His
NELL2$^{\text{EGF1-6 R452A}}$	Polyhedrin	Mouse	G397-T637	C-terminal His
NELL2$^{\text{EGF1-6 D477A-Y478A}}$	Polyhedrin	Mouse	G397-T637	C-terminal His
NELL2$^{\text{EGF1-6 L498A-F500A}}$	Polyhedrin	Mouse	G397-T637	C-terminal His
NELL2$^{\text{EGF1-6 V509A}}$	Polyhedrin	Mouse	G397-T637	C-terminal His
NELL1$^{\text{NELL2-EGF1-3}}$	Polyhedrin	Mouse	NELL1 D25-R390, NELL2 G397-K522, NELL1 A517-N809	C-terminal His
NELL2$^{\text{NELL1-EGF1-3}}$	Polyhedrin	Mouse	NELL2 G23-K396, NELL1 G391-K516, NELL2 A523-L816	C-terminal His
Robo3^{ECD}	Polyhedrin	Mouse	D22-K890	C-terminal His
Robo3$^{\text{FN1-3}}$	Polyhedrin	Mouse	G551-L864	C-terminal Avi and His tags
Robo1^{ECD}	Polyhedrin	Mouse	R30-S840	C-terminal His
Robo1^{ECD}	Polyhedrin	Mouse	R30-S840	C-terminal Avi and His tags
Robo1$^{\text{FN1-3}}$	Polyhedrin	Mouse	N519-S840	C-terminal Avi and His tags
Robo1$^{\text{Robo3-FN1}}$	Polyhedrin	Mouse	Robo1 R30-V510, Robo3 S545-P657, Robo1 V618-S840	C-terminal Avi and His tags
NELL2^{FL}	Polyhedrin	Human	L22-L816	C-terminal His
NELL2$^{\Delta\text{CC}}$	Polyhedrin	Human	L22-G236, E271-L816	C-terminal His
NELL2$^{\text{EGF1-6}}$	Polyhedrin	Human	G397-T637	C-terminal His
NELL2$^{\text{EGF1-3}}$	Polyhedrin	Human	G397-A523	C-terminal His
NELL1$^{\text{EGF1-3}}$	Polyhedrin	Human	R390-A517	C-terminal His
Robo3$^{\text{FN1}}$	Polyhedrin	Human	T550-S562	C-terminal His
Robo3$^{\text{FN1-3}}$	Polyhedrin	Human	P549-L870	C-terminal His
Robo1$^{\text{FN1-3}}$	Polyhedrin	Human	N522-L875	C-terminal Avi and His tags

Supplementary Movie 1. Movie of a commissural axon in a NELL2 gradient. 2-h time-lapse movie of a wild-type commissural neuron (shown in Fig. 1C) responding to a NELL2 gradient with 50 ng/ml peak concentration. Gradient direction is top/high to bottom/low. The axon turns away from the source of NELL2.

Supplementary Movie 2. Movie of a *Robo3*$^{-/-}$ in a NELL2 gradient. 2-h time-lapse movie of a *Robo3*$^{-/-}$ commissural neuron (shown in Fig. 1E) responding to a NELL2 gradient with 50 ng/ml peak concentration. Gradient direction is top/high to bottom/low. The axon fails to turn away from the source of NELL2.
