# Supplementary material for: NELL2-Robo3 complex structure reveals mechanisms of receptor activation for axon guidance
Source: Nat Commun. 2020 Mar 20;11:1489. doi: 10.1038/s41467-020-15211-1 (PMC7083938; doi:10.1038/s41467-020-15211-1)
Supplement: Supplementary file 3 — Description of Additional Supplementary Files [file 41467_2020_15211_MOESM3_ESM.pdf]

Description of Additional Supplementary Files

File Name: Supplementary Movie 1
Description: Movie of a commissural axon in a NELL2 gradient. 2-h time-lapse movie of a wild-type commissural neuron (shown in Fig. 1c) responding to a NELL2 gradient with50 ng/ml peak concentration. Gradient direction is top/high to bottom/low. The axon turns away from the source of NELL2.

File Name: Supplementary Movie 2
Description: Movie of a Robo3-/- in a NELL2 gradient. 2-h time-lapse movie of a Robo3-/- commissural neuron (shown in Fig. 1e) responding to a NELL2 gradient with 50 ng/ml peak concentration. Gradient direction is top/high to bottom/low. The axon fails to turn away from the source of NELL2.
