# Supplementary material for: NELL2-Robo3 complex structure reveals mechanisms of receptor activation for axon guidance
Source: Nat Commun. 2020 Mar 20;11:1489. doi: 10.1038/s41467-020-15211-1 (PMC7083938; doi:10.1038/s41467-020-15211-1)
Supplement: Supplementary file 6 — Reporting Summary [file 41467_2020_15211_MOESM6_ESM.pdf]

nature research

Corresponding author(s): Alexander Jaworski, Engin Ozkan

Last updated by author(s): Jan 2, 2020

# Reporting Summary

Nature Research wishes to improve the reproducibility of the work that we publish. This form provides structure for consistency and transparency in reporting. For further information on Nature Research policies, see Authors & Referees and the Editorial Policy Checklist .

## Statistics

For all statistical analyses, confirm that the following items are present in the figure legend, table legend, main text, or Methods section.

| n/a | Confirmed | |
|---|---|---|
| ☐ | ☒ | The exact sample size (*n*) for each experimental group/condition, given as a discrete number and unit of measurement |
| ☐ | ☒ | A statement on whether measurements were taken from distinct samples or whether the same sample was measured repeatedly |
| ☐ | ☒ | The statistical test(s) used AND whether they are one- or two-sided<br>*Only common tests should be described solely by name; describe more complex techniques in the Methods section.* |
| ☒ | ☐ | A description of all covariates tested |
| ☒ | ☐ | A description of any assumptions or corrections, such as tests of normality and adjustment for multiple comparisons |
| ☐ | ☒ | A full description of the statistical parameters including central tendency (e.g. means) or other basic estimates (e.g. regression coefficient) AND variation (e.g. standard deviation) or associated estimates of uncertainty (e.g. confidence intervals) |
| ☐ | ☒ | For null hypothesis testing, the test statistic (e.g. *F*, *t*, *r*) with confidence intervals, effect sizes, degrees of freedom and *P* value noted<br>*Give P values as exact values whenever suitable.* |
| ☒ | ☐ | For Bayesian analysis, information on the choice of priors and Markov chain Monte Carlo settings |
| ☒ | ☐ | For hierarchical and complex designs, identification of the appropriate level for tests and full reporting of outcomes |
| ☒ | ☐ | Estimates of effect sizes (e.g. Cohen's *d*, Pearson's *r*), indicating how they were calculated |

*Our web collection on statistics for biologists contains articles on many of the points above.*

## Software and code

Policy information about availability of computer code

| | |
|---|---|
| Data collection | For immunofloresence and DIC imaging:<br>Nikon Eclipse Ti inverted microscope with Andor CCD camera - Nikon NIS Software - version 4.50 or later |
| Data analysis | Crystallographic software used: XDS (version 20170601), PHENIX (version: 1.14-3260).<br>Prism for protein fitting binding data (Langmuir isotherms): Prism (version 6c)<br>ImageJ 1.15s - NIH, USA (Java 1.80_101 (64 bit))<br>Nikon NIS Software - version 4.50 or later<br>Graphpad Prism - version 8<br>Windows Excel 2018 - version 16.16.5<br>Dunn chamber analysis Macro for ImageJ: Provided by Charron Lab (Yam et al, 2009) |

For manuscripts utilizing custom algorithms or software that are central to the research but not yet described in published literature, software must be made available to editors/reviewers. We strongly encourage code deposition in a community repository (e.g. GitHub). See the Nature Research guidelines for submitting code & software for further information.

## Data

Policy information about availability of data

All manuscripts must include a data availability statement. This statement should provide the following information, where applicable:

- Accession codes, unique identifiers, or web links for publicly available datasets
- A list of figures that have associated raw data
- A description of any restrictions on data availability

The datasets generated during and/or analyzed during the current study are available from the corresponding author on reasonable request.

# Field-specific reporting

Please select the one below that is the best fit for your research. If you are not sure, read the appropriate sections before making your selection.

☒ Life sciences ☐ Behavioural & social sciences ☐ Ecological, evolutionary & environmental sciences

For a reference copy of the document with all sections, see nature.com/documents/nr-reporting-summary-flat.pdf

# Life sciences study design

All studies must disclose on these points even when the disclosure is negative.

| | |
|---|---|
| Sample size | For in vitro experiments, between 3-5 embryos were used for each experimental condition. Between 25-100 neurons were analyzed per embryo. Sample sizes were determined by the current standard used for mice in cellular and molecular neuroscience experiments, based on the minimal amount of mice required to detect significance with an alpha rate set at .05 in a standardly powered experiment. |
| Data exclusions | No data was excluded from analysis |
| Replication | All in vitro experiments were performed over 3-5 independent replicates with each embryo treated as a single n. Cell-based binding experiments were performed in duplicate. |
| Randomization | Organisms were organized by genotype for all experiments. Experiments would always be conducted in parallel across all genotypes to reduce variation. |
| Blinding | No blinding was performed for the analysis of cell-based binding experiments, as there is no way to introduce bias into this experiment. Analysis of the axon turning assay is intrinsically blind, as measurements of each axon are taken while naive to the direction of the cue gradient, as well as identity of the neuron being analyzed. |

# Reporting for specific materials, systems and methods

We require information from authors about some types of materials, experimental systems and methods used in many studies. Here, indicate whether each material, system or method listed is relevant to your study. If you are not sure if a list item applies to your research, read the appropriate section before selecting a response.

## Materials & experimental systems

| n/a | Involved in the study | |
|---|---|---|
| ☐ | ☒ | Antibodies |
| ☐ | ☒ | Eukaryotic cell lines |
| ☒ | ☐ | Palaeontology |
| ☐ | ☒ | Animals and other organisms |
| ☒ | ☐ | Human research participants |
| ☒ | ☐ | Clinical data |

## Methods

| n/a | Involved in the study | |
|---|---|---|
| ☒ | ☐ | ChIP-seq |
| ☐ | ☒ | Flow cytometry |
| ☒ | ☐ | MRI-based neuroimaging |

## Antibodies

| | |
|---|---|
| Antibodies used | Goat polyclonal anti-Robo3 (R&D Systems, AF3076)<br>Goat polyclonal anti-TAG1 (R&D Systems, AF4439)<br>Rabbit polyclonal anti-NELL2 (Nakamoto Lab, Jiang et al. 2009)<br>Rabbit polyclonal anti-RFP (Rockland, 600-401-379)<br>Rabbit polyclonal anti-TuJ1 (Biolegend, 802001)<br>Rabbit monoclonal anti-Robo3.1 (Tessier-Lavigne Lab, Chen et al. 2008). |
| Validation | Robo3 (R&D Systems) - Company reports antibody detects human and mouse ROBO3 in direct ELISAs and Western blots. We independently verified specificity of this antibody, there was an absolute absence of signal in Robo3 null mouse tissue.<br><br>TAG1 (R&D Systems) - Company reports antibody detects mouse and rat Contactin-2 in direct ELISAs, Western blots, and immunohistochemistry. We independently verified specificity of the antibody, there was an absolute absence of signal in TAG-1 knockout mouse tissue.<br><br>NELL2 (Nakamoto Lab) - Antibody was verified previously by the Nakamoto lab (Jiang et al, 2009). We independently verified specificity of the antibody, there was an absolute absence of signal in NELL2 knockout mouse tissue.<br><br>RFP (Rockland) - This antibody has been cited in 165 publications. We independently verified specificity of the antibody, there |

was an absolute absence of signal in cells that had not been forced to express RFP.

TuJ1 (Biolegend) - Supplier reports this antibody is well characterized and highly reactive to neuron-specific Class III Beta-tubulin. TuJ1 does not identify tubulin found in glial cells. TuJ1 recognizes an epitope located within the last 15 C-terminal residues- cited over 12 times and has been well characterized previously by other laboratories.

Robo3.1 (Tessier-Lavigne Lab) - Antibody was verified previously by the Tessier-Lavigne lab (Chen et al, 2008). We independently verified specificity of the antibody, there was an absolute absence of signal in Robo3 null tissue.

# Eukaryotic cell lines

Policy information about cell lines

| | |
|---|---|
| Cell line source(s) | COS-7 fibroblast-like cell line from monkey kidney - Purchased from ATCC - Catalog #CRL-1651<br>HEK293 epithelial-like cell line from human embryonic kidney - Purchased from ATCC - Catalog #CRL-1573<br>High Five cells (BTI-Tn-5B1-4) from Trichoplusia ni - for protein expression using baculoviruses - Thermo Fisher #B855-02 |
| Authentication | COS7 and HEK293 cell lines (from ATCC) have been validated by the supplier through STR analysis and cytogenetic studies. |
| Mycoplasma contamination | Cell lines were not tested for mycoplasma contamination |
| Commonly misidentified lines<br>(See ICLAC register) | none |

# Animals and other organisms

Policy information about studies involving animals; ARRIVE guidelines recommended for reporting animal research

| | |
|---|---|
| Laboratory animals | Robo3 null mice; described and genotyped in Sabatier et al., Cell 2004. All mice were maintained on a CD-1 background. Tissue sections or neuronal cultures were prepared from embryos of either sex. |
| Wild animals | No wild animals were used in this study |
| Field-collected samples | No field-collected samples were used in this study |
| Ethics oversight | Brown University IACUC |

Note that full information on the approval of the study protocol must also be provided in the manuscript.

# Flow Cytometry

## Plots

Confirm that:

- [x] The axis labels state the marker and fluorochrome used (e.g. CD4-FITC).
- [x] The axis scales are clearly visible. Include numbers along axes only for bottom left plot of group (a 'group' is an analysis of identical markers).
- [ ] All plots are contour plots with outliers or pseudocolor plots.
- [ ] A numerical value for number of cells or percentage (with statistics) is provided.

## Methodology

| | |
|---|---|
| Sample preparation | We did not use a biological source. The flow cytometry experiments were done for a binding analysis between two proteins on a biologically irrelevant Drosophila cell line (S2 cells). |
| Instrument | Accuri C6 |
| Software | Accuri/BD Csampler (manufacturer's software). Software does not produce contour plots. |
| Cell population abundance | No sorting was done. |
| Gating strategy | FSC/SSC gate eliminating cellular debris is shown in Suppl Fig 3b. |

- [x] Tick this box to confirm that a figure exemplifying the gating strategy is provided in the Supplementary Information.
